# Spontaneous improvement in randomised clinical trials: meta-analysis of three-armed trials comparing no treatment, placebo and active intervention

**DOI:** 10.1186/1471-2288-9-1

**Published:** 2009-01-05

**Authors:** Lasse Theis Krogsbøll, Asbjørn Hróbjartsson, Peter C Gøtzsche

**Affiliations:** 1 The Nordic Cochrane Centre, Rigshospitalet, Dept. 3343, Blegdamsvej 9, DK-2100 Copenhagen, Denmark

## Abstract

**Background:**

It can be challenging for patients and clinicians to properly interpret a change in the clinical condition after a treatment has been given. It is not known to which extent spontaneous improvement, effect of placebo and effect of active interventions contribute to the observed change from baseline, and we aimed at quantifying these contributions.

**Methods:**

Systematic review and meta-analysis, based on a Cochrane review of the effect of placebo interventions for all clinical conditions. We selected all trials that had randomised the patients to three arms: no treatment, placebo and active intervention, and that had used an outcome that was measured on a continuous scale or on a ranking scale. Clinical conditions that had been studied in less than three trials were excluded.

**Results:**

We analysed 37 trials (2900 patients) that covered 8 clinical conditions. The active interventions were psychological in 17 trials, physical in 15 trials, and pharmacological in 5 trials. Overall, across all conditions and interventions, there was a statistically significant change from baseline in all three arms. The standardized mean difference (SMD) for change from baseline was -0.24 (95% confidence interval -0.36 to -0.12) for no treatment, -0.44 (-0.61 to -0.28) for placebo, and -1.01 (-1.16 to -0.86) for active treatment. Thus, on average, the relative contributions of spontaneous improvement and of placebo to that of the active interventions were 24% and 20%, respectively, but with some uncertainty, as indicated by the confidence intervals for the three SMDs. The conditions that had the most pronounced spontaneous improvement were nausea (45%), smoking (40%), depression (35%), phobia (34%) and acute pain (25%).

**Conclusion:**

Spontaneous improvement and effect of placebo contributed importantly to the observed treatment effect in actively treated patients, but the relative importance of these factors differed according to clinical condition and intervention.

## Background

It can be challenging for patients and clinicians to properly interpret a change in the clinical condition after a treatment has been given. An improvement will often be ascribed to the treatment, although at least two other factors often play a role.

One factor is spontaneous improvement [1]. Many clinical conditions are self-limiting, e.g. headache, acute low back pain and the common cold, and most chronic disease symptoms fluctuate in intensity, e.g. rheumatoid arthritis, chronic low back pain and psoriasis. Patients will often seek medical attention when their symptoms are worst, and they are most likely to be included in randomised trials at this time. For the purpose of this paper, we regarded regression to the mean effects as being part of the spontaneous improvement. Regression to the mean occurs, for example, when a patient can only be included in a trial if the symptoms are worse than some threshold value; for statistical reasons, the value will then likely be lower at a later time [1,2].

The second factor is the effect of placebo. Patients may feel reassured, change their expectation, or re-interpret their symptoms once a treatment has been commenced. A Cochrane systematic review did not find large effects of placebo, but some effect in trials with patient-reported continuous outcomes, especially pain [3-5].

**Figure 1:**
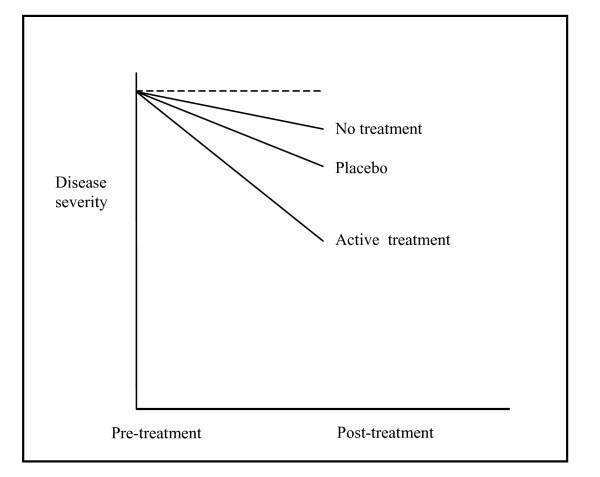
**Illustration of approximate contributions of spontaneous improvement and effect of placebo to the estimated effect of active interventions**.

We have not found any previous reviews of the three main factors affecting the clinical course of patients included in randomised clinical trials: spontaneous improvement, effect of placebos and effect of active interventions (Fig. 1). We aimed at quantifying their relative contribution to change from baseline in randomised trials.

## Methods

The Cochrane review of the effect of placebo interventions involved a thorough search for trials including a no-treatment arm and a placebo arm. We selected all trials from the updated Cochrane review of placebo interventions [5] that had randomised the patients to three arms: no treatment, placebo and active intervention, and that had used an outcome that was measured on a continuous scale or on a ranking scale. In order to permit analyses of separate clinical conditions, we excluded conditions studied in less than three trials.

Potentially eligible trial reports were read in full by one author (LK), who made preliminary decisions on inclusion and choice of outcome, and extracted the data. The authors of the Cochrane review (AH and PCG) checked the selections and the extracted data. Disagreements were resolved by discussion.

In the Cochrane review, patient-reported outcomes were preferred to observer-reported ones. For this study, we selected the outcome that we found most relevant, disregarding whether it was patient- or observer-reported. We made this decision by consensus; there was very little disagreement. In seven cases, the chosen outcome was different from that in the original review. An example is the selection of the well-known observer-reported Bech-Rafaelsen Melancholia Scale instead of the patient-reported Befindlichkeits-Skala.

Data extraction was done using a pilot-tested chart. For each trial, pre- and post-treatment means, standard deviations and group sizes were extracted for the three arms. Additional information extracted was: clinical condition, acute or chronic problem, name and range of scale used, and type of intervention (physical, pharmacological or psychological).

Meta-analysis was done using Comprehensive Meta Analysis [computer program] version 2.2.030, July 2006.

Standardized mean differences (SMD) with 95% confidence intervals were calculated for each trial. SMD is the difference in means divided by the pooled standard deviation. SMD was calculated as Hedges' g, with adjustment for small sample bias. A negative SMD usually implies a positive effect of the intervention, e.g. a lower pain score means less pain. However, in four trials, a large clinical score meant a beneficial effect, and we therefore changed the sign of the SMD before the analysis in these cases. Thus, a negative SMD in our analyses always means a beneficial effect. When standard deviations were missing, we used those from similar trials.

Due to the clinical diversity of the included patients, we did not investigate one treatment effect, but rather the mean of many different treatment effects. There was also substantial methodological heterogeneity, e.g. some trials did not have adequately concealed treatment allocation. We therefore used a random effects model for the analyses. The degree of heterogeneity was investigated with I^2^, which describes the percentage of the variability in effect estimates that is due to heterogeneity rather than sampling error [6].

It was not straightforward how to do the analyses, as we needed to compare the effects in the three groups with the condition at baseline. We analyzed the three treatment arms separately by comparing the post-treatment values with the values at baseline. These data were paired, but we analyzed them as if they were independent, as the presentation of data in the articles did not allow paired analyses. Thus, we accepted a moderate loss of statistical power by handling the paired data as unpaired and assumed that the effect of the ignored correlations between pre- and post-intervention measurements was the same in all situations. It should be noted that this approach leads to overestimation of the sampling error, and therefore to underestimation of the heterogeneity.

It was not possible to determine group sizes both pre- and post-treatment for all trials. We therefore used post-treatment sizes in the analyses, which has the advantage that treatment arms with relatively more dropouts receive less weight.

Ten trials had more than one active treatment. In the meta-analysis, these were entered as separate treatment arms and therefore contributed relatively more than trials with only one active treatment arm. However, the same would occur in trials with skewed randomisation ratios, and overall, the numbers of patients contributing to the results of the three treatment arms were not much different.

## Results

### In- and exclusion of trials

**Figure 2:**
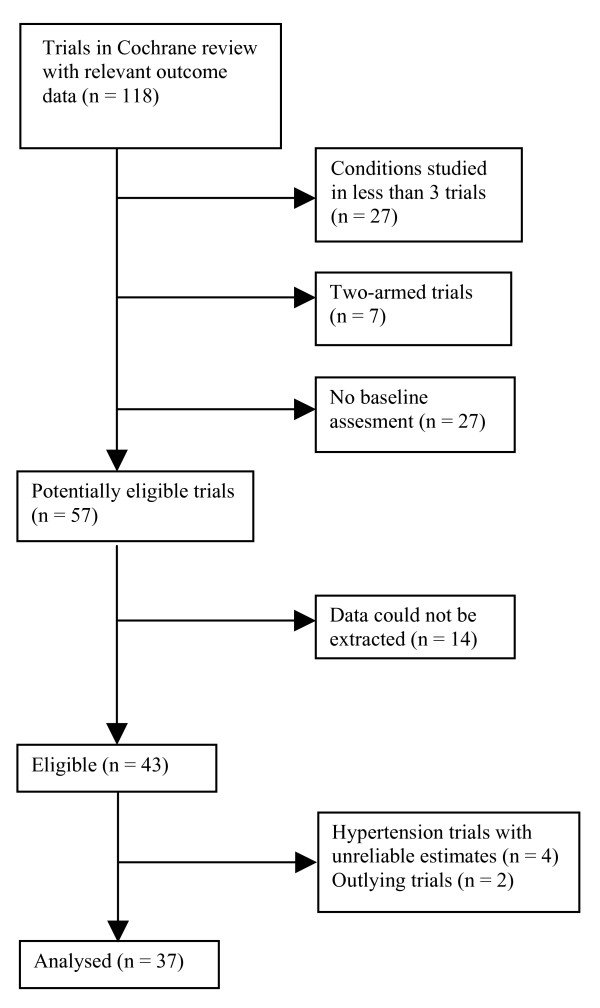
**Selection of trials for the review**.

There were 118 trials in the Cochrane review with continuous outcome data. We excluded 61 trials: seven were two-armed; in 27 trials, the clinical condition had been studied in less than 3 trials; and 27 trials did not have a baseline assessment. Almost all of the trials without a baseline addressed acute conditions, for example acute pain during a procedure. Though such trials often had pre-treatment assessments they did not involve an assessment of pain experienced during the procedure, or the treatment was given before the painful procedure was initiated. Thus, we identified 57 eligible trials. Data necessary for meta-analyses could not be obtained from 14 trials, so we initially included 43 trials [7-49] (Fig. 2).

We found that the estimates for four hypertension trials [46-49] were unreliable for our purpose. Three of the four trials had run-in periods of 4 to 8 weeks before randomisation, which eliminates the regression to the mean effect, and the changes from baseline were therefore very small and unstable.

**Figure 3:**
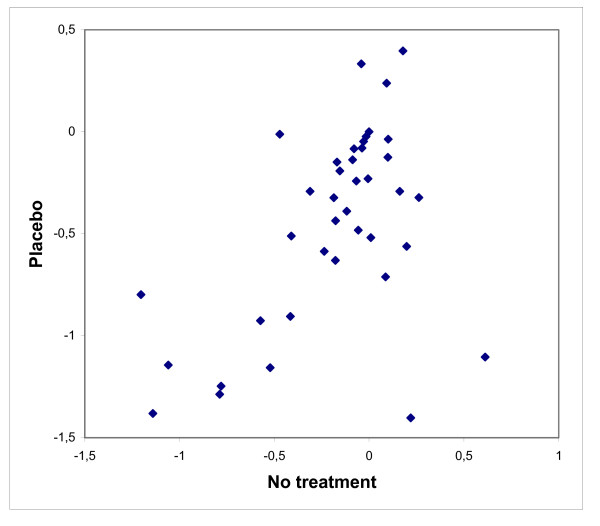
**Change from baseline in the no-treatment and the placebo arms of the 37 analysed trials**. The results are shown as standardized mean differences.

We expected that the change from baseline in the no-treatment arm and the placebo arm would covary from trial to trial, so that when it was large in one arm, it also tended to be large in the other. We verified this, but with two clear outliers (Fig. 3, lower right corner). In one nausea trial [10], the placebo therapy consisted of talks about the child's daily life, which might have had a large reassuring effect. In the other trial [33], the smoking rate was monitored for one week before treatment in the placebo group, but not in the no-treatment group.

Our overall results were very similar, whether or not we excluded the four hypertension trials and the two outliers, but we feel the results for nausea and smoking are more reliable without the outliers. We report below the results for 37 trials (2900 patients), after these six trials were excluded.

### Characteristics of included trials

The 37 trials covered eight different clinical conditions. Most active interventions were of a psychological (17 trials) or physical nature (15 trials); 5 trials were of drugs. Typical psychological treatments were cognitive behaviour therapy and hypnosis, and physical treatment was often acupuncture. Only 10 trials investigated conditions defined by us as acute: depression [7-9], nausea [11,12], and acute pain [13-17], while 27 trials investigated chronic conditions: chronic pain [18-28], phobia [29-31], smoking [32,34], obesity [35-39] and insomnia [40-45]. Duration of treatment was highly variable, ranging from a few days to several months. The outcome was patient-reported in 26 trials and observer-reported in 11 trials.

### Statistical analyses

**Figure 4:**
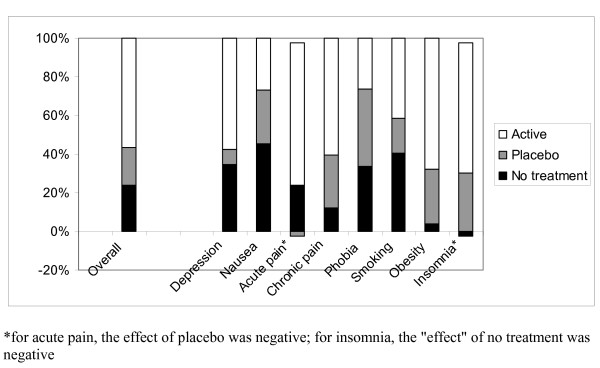
**Relative contributions of the spontaneous improvement, effect of placebo, and effect of active treatment to the change from baseline seen in the actively treated group**.

Overall, across all conditions and interventions, there was a statistically significant change from baseline in all three arms (Table 1). The SMD was -0.24 (95% confidence interval -0.36 to -0.12, I^2 ^= 25%) for no treatment, -0.44 (-0.61 to -0.28, I^2 ^= 57%) for placebo, and -1.01 (-1.16 to -0.86, I^2 ^= 57%) for active treatment. Thus, on average, the relative contributions of spontaneous improvement and of placebo to the change from baseline in the active intervention groups were 24% (0.24/1.01) and 20% ((0.44-0.24)/1.01), respectively (shown approximately in Fig. 1), but with wide variation related to the studied clinical conditions and interventions (Fig. 4). The most pronounced spontaneous improvements, relative to the change from baseline in the actively treated groups, were seen in nausea 45%, smoking 40%, depression 35%, phobia 34% and acute pain 25% (Fig. 4). When combining the influence of spontaneous remission and placebo, the similar proportions were for nausea 73%, smoking 59%, depression 43%, phobia 74%, and acute pain 23% (Fig. 4).

**Table 1:** Standardized mean differences (SMD) for changes from baseline in the three treatment arms separately.

	No. of patients	No. of treatment arms	SMD (95% confidence interval)	I^2 ^(%)
**No treatment Overall**	**978**	**37**	**-0.24 (-0.36 to -0.12)**	**25**

Depression	42	3	-0.44 (-1.27 to 0.39)	70
Nausea	60	2	-0.63 (-0.97 to -0.30)	0
Pain – acute	63	5	-0.53 (-1.03 to -0.02)	49
Pain – chronic	265	11	-0.10 (-0.27 to 0.06)	0
Phobia	28	3	-0.39 (-0.91 to 0.12)	3
Smoking	391	2	-0.40 (-0.54 to 0.26)	0
Obesity	46	5	-0.02 (-0.41 to 0.36)	0
Insomnia	83	6	0.03 (-0.27 to 0.33)	0
				
**Placebo Overall**	**849**	**37**	**-0.44 (-0.61 to -0.28)**	**57**

Depression	44	3	-0.54 (-1.53 to 0.44)	80
Nausea	59	2	-1.02 (-1.37 to -0.66)	0
Pain – acute	62	5	-0.48 (-1.03 to 0.07)	57
Pain – chronic	241	11	-0.32 (-0.59 to -0.04)	54
Phobia	32	3	-0.85 (-1.35 to -0.36)	0
Smoking	272	2	-0.58 (-1.37 to 0.22)	78
Obesity	58	5	-0.17 (-0.52 to 0.18)	0
Insomnia	81	6	-0.31 (-0.61 to 0.00)	0
				
**Active treatment Overall**	**1073**	**52**	**-1.01 (-1.16 to -0.86)**	**57**

Depression	49	3	-1.27 (-1.91 to -0.62)	53
Nausea	60	2	-1.39 (-2.58 to -0.23)	87
Pain – acute	61	5	-2.12 (-3.01 to -1.24)	73
Pain – chronic	373	17	-0.81 (-1.03 to -0.59)	52
Phobia	48	5	-1.15 (-1.67 to -0.63)	33
Smoking	275	3	-0.99 (-1.16 to -0.81)	0
Obesity	61	5	-0.53 (-1.23 to 0.17)	73
Insomnia	146	12	-1.06 (-1.30 to -0.82)	0

The point estimates were very similar in trials with patient-reported and observer-reported outcomes (Table 2) whereas trials involving acute conditions tended to have larger improvements in all three arms compared with trials involving chronic conditions (Table 3), as expected.

**Table 2:** Standardized mean differences (SMD) for changes from baseline grouped by patient- and observer-reported outcome.

	No. of treatment arms	SMD (95% confidence interval)	I^2 ^(%)
**No treatment**			

Observer-reported	11	-0.28 (-0.56 to -0.01)	20
Patient-reported	26	-0.22 (-0.36 to -0.09)	30
			
**Placebo**			

Observer-reported	11	-0.50 (-0.81 to -0.20)	40
Patient-reported	26	-0.42 (-0.62 to -0.22)	63
			
**Active treatment**			

Observer-reported	16	-0.92 (-1.22 to -0.62)	56
Patient-reported	36	-1.04 (-1.22 to -0.87)	59

**Table 3:** Standardized mean differences (SMD) for changes from baseline grouped by acute or chronic condition.

	No. of treatment arms	SMD (95% confidence Interval)	I^2 ^(%)
**No treatment**			

Acute	10	-0.56 (-0.85 to -0.26)	41
Chronic	27	-0.24 (-0.33 to -0.14)	0
			
**Placebo**			

Acute	10	-0.65 (-1.03 to -0.27)	65
Chronic	27	-0.37(-0.55 to -0.19)	54
			
**Active treatment**			

Acute	10	-1.63 (-2.11 to -1.15)	73
Chronic	42	-0.88 (-1.03 to -0.74)	45

## Discussion

We found that both the spontaneous improvement and the effect of placebo contributed importantly to the observed treatment effect in actively treated patients. As noted above, we have not found other reviews that describe the relative contributions of spontaneous remission and placebo to the improvement clinicians note when they treat patients.

Our findings have two implications. First, they underline that it is a fallacy when patients and clinicians interpret an improvement that occurs after a treatment has been instituted as being caused by that treatment. In fact, we found that, on average, only about half of that improvement could be ascribed to the treatment in the trials we analysed.

Second, our findings show that it is wrong to describe the effect that is observed in a placebo arm of a randomised trial as the effect of placebo, as it includes the spontaneous improvement that would also have occurred without administration of a placebo [50]. This error is very common. We did a full-text search on "placebo effect" on the BMJ's website on 30 April 2008 and found the error in 90% of the articles, even in an obituary.

It is a limitation of our study that a quarter of the eligible trials did not report the data necessary for our meta-analyses. Furthermore, we had to use an unconventional meta-analytic method but find it reassuring that the overall effect of placebo was -0.28, as this agrees closely with our previous estimate of -0.24 in the Cochrane review [5] where we used standard meta-analytic methods. We would not expect more elaborate methods to yield results that differ importantly from those we have reported here.

We considered other approaches and also did more traditional meta-analyses, comparing treatment arms within each trial after treatment and calculating ratios between the three arms before these ratios were pooled, but as the denominators of the per trial ratios had a distribution that crossed zero, these ratios were very unstable because of "division almost by zero" effects. Furthermore, we could not use this standard approach for the spontaneous improvement, as this required comparison with baseline. We did not try to convert our unpaired analyses into paired ones, as this would have required estimations of correlations that were likely to vary between diseases and interventions.

The relative contributions of spontaneous improvement, effects of placebo, and effects of active treatment to the observed change from baseline varied considerably. The eight clinical conditions we analysed were either psychiatric diseases (depression and phobia), involved a high degree of patient cooperation (smoking and obesity) or involved subjective outcomes (acute and chronic pain, nausea, and insomnia); and the interventions were mostly non-pharmacological. It seems likely that spontaneous improvement is more important in trials that include patients with high symptom scores and that do not implement a placebo run-in period, particularly as the regression to the mean is likely to be more pronounced in such settings.

Our Cochrane review suggested that the effect of placebos is smaller when imitating pharmacological interventions and when outcomes are observer-reported [3-5]. It is therefore likely that the effect of placebo is comparatively less important in drug trials and in trials with observer-reported outcomes. The Cochrane review found a small effect of placebo on pain, which we reproduced in this review for chronic pain, but not for acute pain, possibly because we were unable to include many acute pain trials that provided no baseline data.

The active interventions we included seemed to be quite effective, which is surprising, as most of them were unconventional, and as many trials involved acupuncture. We recently did a systematic review of three-armed acupuncture trials and found a small analgesic effect of acupuncture, compared to placebo acupuncture, that seems to lack clinical relevance and could not be clearly distinguished from bias [51]. The apparent effects we noted of active treatments may therefore to some degree reflect bias, e.g. related to unconcealed allocation of patients and unsuccessful blinding.

A major problem related to the interpretation of the outcomes in no-treatment and placebo groups is the lack of blinding. Blinding is important to reduce reporting bias in experiments with subjective outcomes [52], but it is not possible to blind patients who receive no treatment. The lack of blinding favours placebo [52], as patients were often blinded with respect to placebo and active treatment. Patients in the placebo group may think they receive active treatment, or they may tend to please their doctors by exaggerating the improvement, and conversely, patients in the no-treatment group may tend to view their experiences more negatively, as they may feel deprived of treatment.

## Conclusion

We conclude that both the spontaneous improvement and the effect of placebo contribute importantly to the observed treatment effect in actively treated patients, and that the relative importance of these factors differ according to clinical condition and intervention.

## Competing interests

The authors declare that they have no competing interests.

## Authors' contributions

PCG and AH coined the idea, initiated the project, and wrote the protocol with LK; LK extracted data that were checked by PCG and AH; LK and PCG did the analyses; LK wrote the first draft of the paper, PCG and AH the final version. Guarantors: PCG and AH.

## Funding

No funding.

## Pre-publication history

The pre-publication history for this paper can be accessed here:

http://www.biomedcentral.com/1471-2288/9/1/prepub
